# High-dose pharmaceutical-grade biotin in patients with demyelinating neuropathies: a phase 2b open label, uncontrolled, pilot study

**DOI:** 10.1186/s12883-023-03440-y

**Published:** 2023-10-30

**Authors:** Alain Créange, Emilie Hutin, Frédéric Sedel, Ludivine Le Vigouroux, Jean-Pascal Lefaucheur

**Affiliations:** 1 AP-HP, Hôpital Henri Mondor, Service de Neurologie, 94010 Créteil, France; 2 AP-HP, Hôpital Henri Mondor, CRC SEP Grand Paris Est, 94010 Créteil, France; 3 Université Paris Est Créteil, EA4391, ENT, F-94010 Créteil, France; 4 Laboratoire Analyse Et Restauration du Mouvement, Service de Rééducation Neurolocomotrice, AP-HP, Hôpital Henri Mondor, 94010 Créteil, France; 5 Université Paris Est Créteil, EA 7377, BIOTN, F-94010 Créteil, France; 6 Medday Pharmaceuticals, Paris, France; 7 ATLANSTAT, Rezé, France; 8 AP-HP, Hôpital Henri Mondor, Unité de Neurophysiologie Clinique, 94010 Créteil, France

**Keywords:** Biotin, Peripheral neuropathies, Demyelination, Nerve excitability, Inflammatory neuropathy, CMT neuropathy

## Abstract

**Background:**

We proposed to investigate high-dose pharmaceutical-grade biotin in a population of demyelinating neuropathies of different aetiologies, as a proof-of-concept.

**Methods:**

Phase IIb open label, uncontrolled, single center, pilot study in 15 patients (three groups of five patients) with chronic demyelinating peripheral neuropathy, i.e. chronic inflammatory demyelinating polyradiculoneuropathy, anti-myelin-associated glycoprotein neuropathy and Charcot-Marie-Tooth 1a or 1b. The investigational product was high-dose pharmaceutical-grade biotin (100 mg taken orally three times a day over a maximum of 52 weeks.

The primary endpoint was a 10% relative improvement in 2 of the following 4 electrophysiological variables: motor nerve conduction velocity, distal motor latency, F wave latency, duration of the compound muscle action potential. The secondary endpoints included Overall Neuropathy Limitations Scale (ONLS) score, Medical Research Council (MRC) sum score, Inflammatory Neuropathy Cause and Treatment (INCAT) sensory sum score, 10-m walk test, 6-min walk test, posturography parameters, and nerve excitability variables.

**Results:**

The primary endpoint was reached in one patient. In the full population analysis, some secondary endpoints parameters improved: MRC score, INCAT sensory sum score, 6-min walk distance, strength-duration time constant, and rheobase. There was a positive correlation between the improvement in the 6-min walk distance and the strength-duration time constant. Regarding the safety results, 42 adverse events occurred, of which three were of severe intensity but none was considered as related to the investigational product.

**Conclusions:**

Even if the primary endpoint was not met, administration of high-dose pharmaceutical-grade biotin led to an improvement in various sensory and motor parameters, gait abilities, and nerve excitability parameters. The tolerance of the treatment was satisfactory.

**Trial registration:**

ClinicalTrials.gov Identifier: NCT02967679; date 2016/12/05.

**Supplementary Information:**

The online version contains supplementary material available at 10.1186/s12883-023-03440-y.

## Introduction

Chronic inflammatory demyelinating polyradiculoneuropathy (CIDP), polyneuropathy associated with IgM monoclonal gammopathy with antibodies against myelin-associated glycoprotein (MAG) and Charcot-Marie-Tooth (CMT) 1a and 1b are acquired and genetic neuropathies that alter the myelin sheath as a common feature.

In CIDP, the lesions include myelin sheaths and nodes of Ranvier morphological changes leading to alterations in nerve excitability. These alterations include a decrease in Na + /K + adenosine 5′-triphosphatase (ATPase) pump function, intra-axonal Na + accumulation associated with energetic failure and variable modifications in fast and slow Na + and K + voltage-gated channels [1–4].

Anti-MAG antibodies lead to enlargement of the myelin sheath [5] The duplication of the PMP22 gene in CMT1a patients, and point mutations in the myelin protein 0 (MPZ) gene in CMT1b patients result in their overexpression and in abnormal Schwann cell and myelin differentiation.

Despite different pathophysiological mechanisms, these neuropathies share common electrophysiological features of demyelination. They also share common clinical features, including variable degrees of progressive weakness, fatigability, or sensory deficit in both upper and lower limbs, ataxia, and impaired walking abilities.

Treatment options for CIDP include steroids, intravenous immunoglobulins and plasma exchange. However, the long-term (more than 6 months) benefits of these therapies may remain limited, leaving room for the development of chronic and severe disability. There is no consensus about the best treatment strategy for anti-MAG patients [6]. There is currently no approved treatment for either CMT1a or for CMT1b.

Biotin (or vitamin H) is a ubiquitous water-soluble vitamin that is naturally found in many foods. In mammals, biotin acts as a coenzyme for four important carboxylases involved in key steps of energy metabolism (Krebs cycle) and fatty acid synthesis. High-dose pharmaceutical-grade biotin (hdPB) has been considered a potential therapeutic option in demyelinating disease of the CNS, i.e., secondary multiple sclerosis (MS) [7, 8], although this was recently refuted [9]. Biotin improved clinical and neurophysiological parameters in a small series of patients [10] and decreased pain in an experimental study [11].

Previous experience in three Phase IIb/III clinical research studies in humans has provided evidence of no risk of hdPB (300 mg/day) in adult MS patients [7–9, 12].

We hypothesized that a positive effect of hdPB in demyelinating neuropathies could be associated with (i) an increased energy production in demyelinated nerve fibers that would avoid neurodegeneration and improve neuronal functioning and (ii) a stimulation of myelin repair through activation of the acetyl-CoA carboxylase in Schwann cells.

Considering its previous use in MS, we proposed to study the intake of hdPB in a population of patients with demyelinating neuropathies of different etiologies in a proof of concept/pilot study. To this end, we used clinical scales assessing neurological deficits and function, posturography, and electrophysiological techniques of nerve conduction and excitability studies. We chose a neurophysiological primary endpoint less likely to be influenced by a placebo effect in an open-label, uncontrolled study. Previous studies have shown that, at least in the context of CIDP, neurophysiological criteria have proven to be sensitive to changes induced by therapeutics [13]. Moreover, since the present study focused on the consequences of peripheral nerve demyelination whatever its origin and its clinical results, the objective was to investigate how the treatment could modify the metabolic processes and the dysfunction of nerve fibers whose myelin is impaired.

## Patients and methods

### Patients

**Fig. 1:**
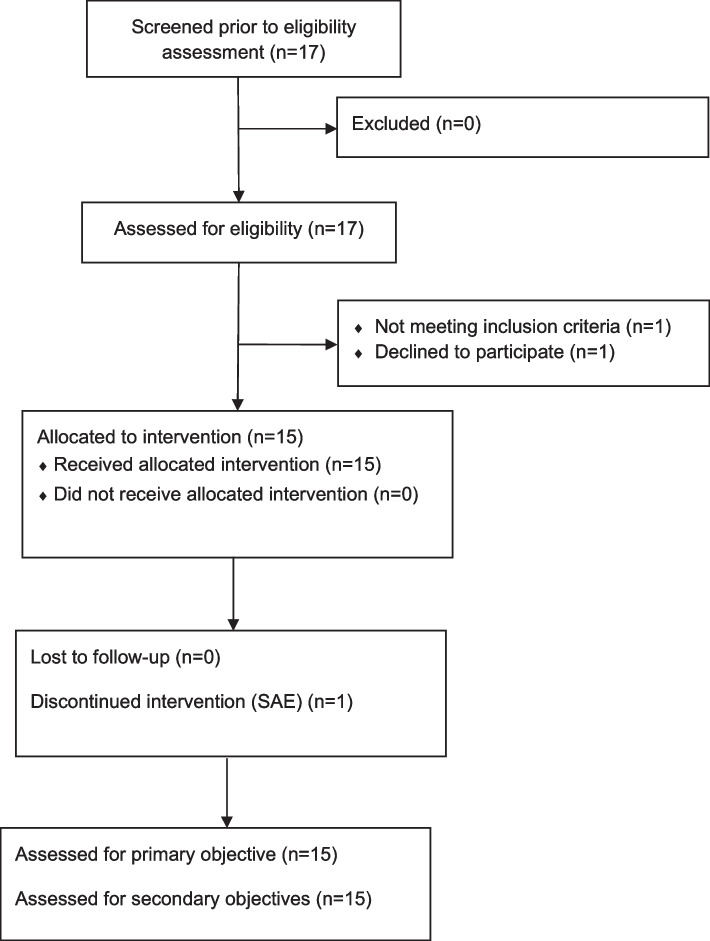
Participant flowchart

This open-label, uncontrolled, single-center, pilot study (ClinicalTrials.gov Identifier: NCT02967679; date 2016/12/05) included 15 patients in 3 distinct groups of 5 patients suffering from specific etiologies of chronic demyelinating peripheral neuropathy: CIDP (*n* = 5), anti-MAG (*n* = 5), CMT1a or CMT1b (*n* = 5) (Fig. 1). The study protocol, amendments, and patient-related documents, were approved by the Independent Ethics Committee (IEC/IRB) of Ile de France VI – Groupe Hospitalier Pitié-Salpêtrière 47, boulevard de l’hôpital 75,013 Paris, France (n° 128–15, Eudra-CT: 2015–001150-15).. Patients were recruited in the Center of Reference for Neuromuscular Disorders of Henri Mondor University Hospital, Créteil, France. The study period ranged from December 05, 2016, to March 18, 2019. The patients fulfilled the diagnostic criteria of either CIDP [14], anti-MAG polyneuropathy, or genetically defined CMT1a or CMT1b, and gave their written informed consent. Other causes of peripheral neuropathies were excluded on the basis of negative results of a large battery of investigations. Absence of neurotoxic drug intake was also checked. Disease-modifying treatment (intravenous immunoglobulins for the 5 patients with CIDP) and symptomatic treatments were not modified during a six-month period preceding the study and during the study period. No medical or surgical history could interfere with the study investigations. No previous or concomitant treatment was considered to interfere with the results of the study.

The main non inclusion criteria consisted of pregnancy, women of childbearing potential without effective contraception, and relapse in the past 3 months before inclusion for CIDP patients.

### Treatment

Capsules of hdPB consisting of 100 mg biotin and excipients (lactose, magnesium stearate, croscarmellose sodium, silica) (MD1003, Medday Pharmaceutical) were taken orally three times a day (one in the morning, one at noon, and one in the evening) over a maximum of 52 weeks.

### Study schedule and procedures

The study schedule consisted of screening and inclusion visits, which were merged, followed by 4 visits at three months intervals over 52 weeks, whereas the initial schedule planned for visits every 12 weeks over 48 weeks. Clinical and functional data were obtained every 12 weeks (baseline W0, W12, W24, W36, W48). Neurophysiological examination, postural stability, and biological testing were performed every 24 weeks (W0, W24, W48).

### Clinical information

The following information was recorded: (i) clinical data (demographic data, history of the patient’s disease, other relevant medical history and ongoing concomitant diseases, clinical examination, vital signs: systolic and diastolic blood pressure, temperature, heart rate, concomitant therapies); (ii) clinical scores (Medical Research Council (MRC) sum score including 19 muscles evaluated bilaterally (max: 190), Inflammatory Neuropathy Cause and Treatment (INCAT) sensory sum score) and functional assessment (timed 10-m walk test, 6-min walk test, Overall Neuropathy Limitations Scale (ONLS) score) [15–17].

### Neurophysiological examination

A conventional motor nerve conduction study was performed as follows: two motor nerves were explored at each upper limb (median and ulnar nerves bilaterally) and each lower limb (peroneal and tibial nerves bilaterally); compound muscle action potentials were recorded at both hands and feet, and also at the leg (tibialis anterior muscle) for the peroneal nerve; two or three sensory nerves were explored at each upper limb (median, ulnar, and radial nerves bilaterally) and each lower limb (peroneal and sural nerves bilaterally). The recorded parameters were: amplitude (peak-to-peak) and duration (negative peak) of the compound muscle action potentials, distal motor latency, motor nerve conduction velocity, and F wave latency for motor nerves, amplitude of sensory nerve action potentials and sensory nerve conduction velocity for sensory nerves. A minimal amplitude was set at 100 µV for compound muscle action potentials and 1 µV for sensory nerve action potentials. Below these values, the potentials were considered as absent (0 µV). The same protocol was applied at each visit for each patient.

In the same session, a protocol of nerve excitability testing was also performed, including the measurement of the following variables: minimal and maximal absolute refractory period duration (ms), refractoriness (%), supernormality (%), strength-duration time constant (SDTC) (ms), and rheobase (mA).

The minimal and maximal absolute refractory period durations were measured according to the double-collision method [18]. The percentages of refractoriness and supernormality were calculated at interstimulus intervals of 2 ms and 7 ms, respectively [19]. Strength–duration properties were determined by measuring the threshold intensities required to obtain 70% Mmax at 0.1-ms and 1.0-ms pulse durations. The strength–duration time constant (chronaxie) and rheobase were calculated using formulae derived from Weiss’s Law [20].

### Spatial–temporal walking analysis

All patients were asked to walk 10 m, barefoot without assistance, on a pressure sensitive carpet (GaitRite Platinum, size 0.61*7.92 m, sample frequency 60 Hz, CIR Systems, Inc., Sparta, NJ, USA), twice at their comfortable and twice at maximal walking velocity. Eight key parameters were calculated over the 7.92 m in the middle of the walkway (excluding the acceleration and deceleration phases) in each condition: mean of speed (m/s), cadence (step/s), step length (m), coefficient of variation of step length, spatial asymmetry index [21], duration of foot support in percentage of the walking cycle (%), step width (m), and foot angle *versus* walking axis (°).

### Postural stability

Participants stood upright barefoot on a force plate (size 400*600 mm, sample frequency 1000 Hz, Bertec Corporation, Columbus, OH, USA) during 60 s in four conditions: eyes open and eyes closed, in 2 standardized positions: feet apart (distance between the calcaneus, 16 cm; angle of the foot, 17°) [22] and feet together. The following dependent variables were extracted from the center of pressure (COP) trajectories: the range of COP positions (i.e. the difference between maximum and minimum COP positions) computed in both anteroposterior (AP range, mm) and medio-lateral directions (ML range, mm); the ellipse best fitting the planar COP data and covering 95% of COP positions (Area, mm^2^) calculated based on a principal component analysis that determined the alignment of its major and minor axes; the mean planar velocity (Velocity, mm/s) calculated as the sum of Euclidian distances between successive positions (total length) divided by the sample duration [23].

### Biological sampling

Biological investigation included a battery of safety and etiological parameters at the time of inclusion (see Online Only Supplements). For women of childbearing potential: a highly sensitive pregnancy test (human chorionic gonadotropin HCG) was performed.

### Evaluation criteria

#### Primary endpoint

The primary endpoint was determined on four motor nerve conduction study variables: motor nerve conduction velocity, distal motor latency, F wave latency, and compound muscle action potential duration. The percent of improvement from baseline to the end of the study (W48) was calculated for each neurophysiological variables and for each of the 8 explored motor nerves (median, ulnar, peroneal, and tibial nerves bilaterally). An improvement ≥ 10% for at least 3 of the 8 explored nerves was arbitrary considered relevant. The primary endpoint was met in a given patient if such a 10% improvement was achieved in at least 2 of the 4 motor nerve conduction study variables.. We assumed that such a change could be considered as really significant and meaningful.

#### Secondary endpoints

Changes from W0 to W48 in the following clinical and electrophysiological parameters:

Clinical endpoints: ONLS score, MRC sum score and total score, INCAT Sensory Sum Score, timed 10-m walk test (s), 6-min walk test distance (m), postural stability parameters and spatial–temporal walking parameters.

Nerve excitability endpoints: minimal and maximal absolute refractory period duration (ms), refractoriness (%), supernormality (%), SDTC (ms), and rheobase (mA).

#### Safety criteria

Analysis of extent of exposure, treatment-emergent adverse events (TEAEs) and laboratory testing.

#### Statistical analysis

The study included 15 patients in total divided into 3 subgroups of 5 patients suffering from either CIDP, anti-MAG or CMT1a/CMT1b. It was arbitrary assumed that, in a pilot proof-of-concept study, 15 patients were a sufficient sample to detect clinical or electrophysiological changes related to hdPB intake or to show the best endpoints for possible future confirmatory studies in the context of the most common types of chronic demyelinating peripheral neuropathies.

The primary analysis of the primary endpoint was evaluated on the full analysis set (FAS) population. Missing values at W48 for the four criteria of demyelination were imputed using the last observation carried forward (LOCF) method. The same analysis as the sensitivity analysis was performed on the per-protocol set population. As a secondary analysis of the primary endpoint, a Wilcoxon signed rank test was performed on the four criteria of demyelination to assess the significance of the relative change from W0 to W48 on the FAS, considering all nerves pooled per patient and criterion. Each test was performed at a 2-sided significance level of 5%. A Wilcoxon signed rank test was performed overall for each parameter to assess whether the absolute changes from W0 were significant at W48. Secondary endpoints were evaluated based on the absolute change from W0 to W48 by disease groups on the FAS. Missing values at W48 were imputed using the LOCF method. A Wilcoxon signed rank test was performed overall for each parameter to assess whether the absolute changes from W0 were significant at W48. We used a Pearson test, after checking the normal distribution of values by the Kolmogorov–Smirnov test, to study the correlation concerning the significant changes observed from W0 to W48 between clinical and neurophysiological data.

## Results

### Patients

All 15 patients allocated to treatment were included in the FAS and safety populations. Seven participants (47%) presented major deviations and were excluded from the per-protocol set population: 3 in the CIDP group, 3 in the anti-MAG group and 1 in the CMT1 group. Major deviations were as follows: compliance not between 80 and 120% (1 patient with anti-MAG); patients without electrophysiological parameters worsening for the past 3 years (7 patients: CIDP, *n* = 3, anti-MAG, *n* = 3 and CMT1, *n* = 1); missing evaluation at W0 or at W48 for motor nerve conduction parameters (1 patient with CIDP); hdPB (investigation medical product) administration duration different from 336 ± 15 days (1 patient with anti-MAG); hdPB administration interruption > 10 consecutive days (1 patient with anti-MAG on his own decision; see supplementary material eTable 1). Demographic and clinical data are presented in Table 1.

**Table 1:** Demographic characteristics and history of the disease-FAS population

Group	CIDP	anti-MAG	CMT1	All
Participants (n)	5	5	5	15
Men (n)	2 (40%)	3 (60%)	2 (40%)	7 (47%)
Women (n)	3 (60%)	2 (40%)	3 (60%)	8 (53%)
Age (years)	66 ± 15; 63 (61;79)	69 ± 8; 65 (63;72)	49 ± 11; 52 (49;55)	61 ± 14; 62 (52;72)
Time since first appearance of symptoms (years)	18 ± 9; 14 (13;15)	11 ± 11; 5 (3;17)	11 ± 16; 5 (3;6)	13 ± 12; 12 (3;17)
Patients with progression (n)	2	2	4	8
Time since progression (years)	2.7 ± 1.5; 1.2 (1.0;1.3)	1.9 ± 1.4; 1.9 (0.7;3.0)	1.6 ± 1.4; 1.4 (0.6;2.5)	1.7 ± 1.3; 1.3 (1.0;2.4)

Age, time since first appearance of symptoms and time since progression are expressed as mean ± SD; median (IQR). If the day and the month of date of the first appearance of symptoms/date of progression were missing, it was estimated by the 15th of June, if only the day was missing, the day was estimated by the 15th of the corresponding month

### Efficacy results

The primary endpoint (primary analysis) was reached in the FAS in 1 (CMT1) patient. This was confirmed in the per-protocol set population. More precisely, in the FAS population, improvements of at least 10% in at least 3 out of 8 nerves were observed in motor nerve conduction velocity in 2 patients, distal motor latency in 4 patients, F wave latency in 0 patients, and compound muscle action potential duration in 4 patients (Tables 2 and 3).

**Table 2:** Nerve conduction study variables. Primary endpoint – Primary analysis

Group	n	10% Improvement for at least 3 motor nerves	Motor Nerve Conduction Velocity (m/s)	Distal Motor Latency (ms)	F Wave Latency (ms)	Duration of the Compound Muscle Action Potential (ms)	Meeting Primary endpoint (change of 2 out of 4 variables)
All	15	No	13 (87%)	11 (73%)	15 (100%)	11 (73%)	14 (93%)
		Yes	2 (13%)	4 (27%)	0	4 (27%)	1 (7%)
CIDP	5	No	4 (80%)	4 (80%)	5 (100%)	4 (80%)	5 (100%)
		Yes	1 (20%)	1 (20%)	0	1 (20%)	0
anti-MAG	5	No	5 (100%)	3 (60%)	5 (100%)	5 (100%)	5 (100%)
		Yes	0	2 (40%)	0	0	0
CMT1	5	No	4 (80%)	4 (80%)	5 (100%)	2 (40%)	4 (80%)
		Yes	1 (20%)	1 (20%)	0	3 (60%)	1 (20%)

Number of patients (%) with at least 10% improvement at W48 for at least 3 of the 8 explored motor nerves for each variable of motor nerve conduction considered in the primary endpoint, which was met (last column) if such a 10% improvement was achieved in at least 2 of the 4 motor nerve conduction study variables. Missing data at W48 (for patients #04 and #09) were imputed with the LOCF method

**Table 3:** Nerve conduction study variables. Secondary analysis

Nerve conduction study variables	n	W0	W48	Relative change between W0 and W48	*p*-value
Motor Nerve Conduction Velocity (m/s)	15	38.5 (19.4;42.0)	35.0 (21.7;41.2)	0.6 (-7.0;7.2)	.762
Distal Motor Latency (ms)	15	6.5 (4.2;8.4)	6.1 (4.1; 8.3)	0.5 (-3.4;4.6)	.600
F Wave Latency (ms)	9	37.3 (34.5;39.3)	41.6 (34.1;46.6)	2.6 (-1.1;12.0)	.164
Duration of the Compound Muscle Action Potential (ms)	15	7.3 (5.7;9.4)	7.8 (5.8;9.7)	2.5 (-0.0;15.6)	.035

Neurophysiological data considering all nerves pooled per patient – FAS population. Missing data at W48 were imputed with the LOCF method (W36 and W24 for patients #04 and #09, CIDP and CMT1 groups, respectively). The *P*-value was calculated with the Wilcoxon signed rank test. The results are expressed as median (IQR)

In the overall FAS population, some noteworthy secondary endpoint parameters showed improvements, as described below. All results are expressed as median interquartile ranges (IQR) (1^st^ quartile;3^rd^ quartile).

### Clinical parameters

The MRC total score increased from 168.0 (160.0;177.0) at W0 to 176.0 (166.0;180.0) at W48 (absolute change, 3.0 (0.0;9.0), *p* = 0.0371), the INCAT sensory sum score decreased from 6.0 (4.0;8.0) at W0 to 3.0 (1.0;7.0) at W48 (absolute change, -3.0 (-3.0;0.0), *p* = 0.0142), and the 6-min walking distance increased from 375.0 (293.0;474.0) m at W0 to 469.0 (278.0;506.0) m at W48 (absolute change, 69.0 (10.0;104.0) m, *p* < 0.001; Tables 4 and 5).

**Table 4:** Clinical scores. Secondary endpoints

	W0	W12	W24	W36	W48	Absolute change between W0 and W48	*p*-value
MRC	168 (160;177)	175 (164;178)	176 (166;179)	178 (164;180)	176 (166;180)	3 (0; 9)	.037
INCAT	6 (4;8)	5 (2;10)	4 (2;7)	4 (2;7)	3 (1;7)	-3 (-3; 0)	.014
Timed 10-m walk (s)	8 (7;12)	7 (6;9)	8 (7;10)	8 (6;12)	7 (6;12)	-1 (-2; 0)	.178
6-min walking distance (m)	375 (293;474)	419 (315;470)	442 (293;489)	427 (285;489)	469 (278;506)	69 (10;104)	< .001
ONLS	3 (2;4)	2 (2;3)	3 (2;3)	3 (2;4)	2 (1;4)	-1 (-1;1)	.264

Missing data at W48 were imputed with the LOCF method (W36 for patient #04, CIDP group). The *P*-value was calculated with the Wilcoxon signed rank test. The results are expressed as median (IQR) calculated in all patients (*n* = 15)

**Table 5:** Six-min walk test. Secondary endpoint

Group	W0	W12	W24	W36	W48	Absolute change between W0 and W48	*p*-value
CIDP	240 (225;300)	268 (238;332)	293 (265;384)	253 (248;360)	248 (226;392)	91 (8;92)	
anti-MAG	435 (375;479)	467 (390;540)	479 (444;489)	432 (422;536)	489 (444;494)	59 (10;69)	
CMT1	439 (375;474)	469 (442;479)	474 (432;511)	478 (434;513)	479 (469;556)	82 (40;104)	
All patients	375 (293;474)	419 (315;470)	442 (293;489)	427 (285;489)	469 (278;506)	69 (10;104)	< .001

Missing data at W48 were imputed with the LOCF method (W36 for patient #04, CIDP group). The results were considered regardless of walking assistance. The results are expressed in meters as median (IQR) in CIDP group (*n* = 5), anti-MAG group (*n* = 5), CMT1 group (*n* = 5) and in all patients (*n* = 15). The *P*-value was calculated with the Wilcoxon signed rank test in all patients (*n* = 15)

### Spatial–temporal walking analysis

Improvement of the walking ability between W0 and W48 was observed regarding cadence from 1.76 (1.66;1.82) step/s at W0 to 1.91 (1.78;1.98) step/s at W48 (absolute change, 0.08 (0.00;0.16) step/s, *p* = 0.0225), duration of right foot support from 0.57 (0.56;0.60) s at W0 to 0.52 (0.50;0.57) s at W48 (absolute change, -0.03 (0.00;0.16) s, *p* = 0.0137) and duration of left foot support from 0.57 (0.54;0.60) s at W0 to 0.53 (0.50;0.56) s at W48 (absolute change, -0.02 (-0.08;0.00) s, *p* = 0.0322) in ‘comfortable speed’ condition.

### Postural stability

The posturography parameters remained unchanged between W0 and W48.

### Excitability parameters

Median (IQR) SDTC values increased from 0.13 (0.12;0.36) ms at W0 to 0.32 (0.14;0.36) ms at W48 (absolute change of 0.05 (0.01;0.14) ms, *p* = 0.039); rheobase values decreased from 12.50 (7.02;20.61) mA at W0 to 7.31 (3.72;11.39) mA at W48 (absolute change of -4.21 (-9.22;-0.78) mA, *p* < 0.001) (Tables 6, 7 and 8).

**Table 6:** Nerve excitability parameters. Secondary endpoints

	W0	W24	W48	Absolute change between W0 and W48	*p*-value
Strength-duration time constant (ms)	0.13 (0.12;0.36)	0.27 (0.12;0.40)	0.32 (0.14;0.36)	0.05 (-0.01;0.14)	.039
Rheobase (mA)	12.50 (7.02;20.61)	10.28 (7.22;18.39)	7.31 (3.72;11.39)	-4.21 (-9.22;-0.78)	< .001
Supernormality (%)	116.0 (101.4;134.9)	128.8 (109.3;139.1)	128.8 (108.1;139.0)	7.0 (-4.3;25.9)	.213
Refractoriness (%)	33.70 (16.50;53.50)	27.10 (0.00;42.80)	28.90 (11.60;42.80)	-15.90 (-24.40;11.60)	.330
Minimum absolute refractory period (ms)	1.30 (1.10;1.62)	1.34 (1.14;1.56)	1.34 (1.14;1.56)	0.00 (-0.10;0.04)	.923
Maximum absolute refractory period (ms)	2.12 (1.70;2.58)	2.02 (1.90;3.20)	2.12 (1.70;2.40)	-0.02 (-0.50;0.40)	.313

Missing data at W48 were imputed with the LOCF method (W24 for patient #04, CIDP group). The *P*-value was calculated with the Wilcoxon signed rank test. The results are expressed as median (IQR) calculated in all patients (*n* = 15)

**Table 7:** Strength-duration time constant. Secondary endpoint

	W0	W24	W48	Absolute change between W0 and W48	*p*-value
CIDP	0.21 (0.13;0.36)	0.17 (0.13;0.36)	0.32 (0.12;0.32)	-0.01 (-0.01;0.00)	
anti-MAG	0.12 (0.11;0.13)	0.27 (0.12; 0.40)	0.26 (0.18;0.33)	0.08 (0.02;0.14)	
CMT1	0.12 (0.12;0.14)	0.31 (0.17; 0.32)	0.39 (0.18;0.40)	0.06 (0.05;0.26)	
All patients	0.13 (0.12;0.36)	0.27 (0.12; 0.40)	0.32 (0.14;0.36)	0.05 (-0.01;0.14)	.039

Missing data at W48 were imputed with the LOCF method (W24 for patient #04, CIDP group). The results are expressed in ms as median (IQR), in CIDP group (*n* = 5), anti-MAG group (*n* = 5), CMT1 group (*n* = 5) and in all patients (*n* = 15). The *P*-value was calculated with the Wilcoxon signed rank test in all patients (*n* = 15)

**Table 8:** Rheobase

	W0	W24	W48	Absolute change between W0 and W48	*p*-value
CIDP	13.28 (5.56;14.61)	10.28 (7.72;15.28)	4.39 (3.72;12.50)	-1.17 (-1.74;-0.78)	
anti-MAG	19.44 (12.39;20.61)	7.22 (7.17; 16.83)	5.38 (4.67;11.22)	-8.22 (-9.22;-7.72)	
CMT1	11.06 (7.23;12.50)	16.83 (7.28;18.39)	7.31 (7.31;10.72)	-4.21 (-5.19;-0.34)	
All patients	12.50 (7.02;20.61)	10.28 (7.22;18.39)	7.31 (3.72;11.39)	-4.21 (-9.22;-0.78)	< .001

Missing data at W48 were imputed with the LOCF method (W24 for patient #04, CIDP group). The results are expressed in mA as median (IQR), in CIDP group (*n* = 5), anti-MAG group (*n* = 5), CMT1 group (*n* = 5) and in all patients (*n* = 15). The *P*-value was calculated with the Wilcoxon signed rank test in all patients (*n* = 15)

### Clinical and neurophysiological correlations

We studied the correlations between the parameters that significantly changed from W0 to W48, i.e. the 6-min walk test for clinical parameters and SDTC and rheobase for electrophysiological parameters. There was a positive correlation between SDTC and walking capacities evaluated by the 6-min walk test (*r* = 0.579; *p* = 0.049) but no correlation between rheobase and the 6-min walk test (*r* = -0.203; *p* = 0.528).

### Safety results

Overall, 14 patients presented 42 TEAEs (eTable 2). Three TEAEs were of severe intensity in 3 patients, including 1 patient in each disease group: a relapsing autoimmune encephalopathy occurred 428 days after hdPB discontinuation in a patient with CIDP, leading to death; one renal clear cell carcinoma occurred in a patient with anti-MAG neuropathy; and left external malleolar fracture occurred in a patient with CMT1. None of these events was considered to be related to hdPB treatment.

Laboratory test interferences due to hdPB were reported in 2 patients, leading to incorrect interpretation of thyroid tests.

All other TEAEs were mild to moderate in intensity (see eTable 2) without relevant differences between disease groups. No clinically relevant shifts in biological parameters were observed during the study.

## Discussion

This phase IIb open-label trial investigating the effect of hdPB in demyelinating peripheral neuropathies did not reach the primary endpoint, which consisted of a composite nerve conduction study criterion. However, several clinical, functional, and neurophysiological secondary endpoints were achieved. Improvements in clinical deficits and disability were observed, as well as changes in nerve excitability parameters, i.e., SDTC (chronaxie) increase and rheobase decrease. Moreover, we found a positive correlation between improvements in the 6-min walk test and the SDTC.

Standard nerve conduction studies did not show any modification in the overall population, whereas excitability parameter changes occurred, regardless the small sample size. These two types of electrophysiological studies do not assess the same aspects of nerve function and certainly do not have the same sensitivity to changes induced by therapy. Nerve conduction study parameters is a surrogate of the propagation of nerve impulses and the structural properties of axons, while nerve excitability parameters are related to physiological properties of axonal membrane potential changes, involving various slow and fast K_V_ channels, slow Na_V_ channels and the Na + /K + ATPase pump [3, 24–26]. Different alterations of these nerve excitability parameters have been reported in demyelinating neuropathies of acquired or genetic origin [3, 27]. Excitability studies are an interesting way to assess drug-induced changes in nerve function. For example, excitability changes have been observed shortly after IVIg and following intravenous immunoglobulin infusions [4, 13].

In this study, the main changes in nerve excitability after hdPB intake were a decrease in rheobase and an increase in chronaxie (SDTC), two variables whose reproducibility has been shown in several studies, including those performed in our laboratory [28–30]. In CIDP, rheobase is known to be increased and SDTC to be decreased [31], while no such changes have been previously reported in patients with anti-MAG neuropathy and CMT1 [32, 33]. Chronaxie and rheobase mainly reflect nodal aspects of resting membrane potential and depend on the amount of persistent Na + currents through the slow Na_V_ channels. A reduced rheobase and increased SDTC, as observed after hdPB treatment, suggest an increased expression or activity of slow Na_V_ channels [34, 35]. An inward persistent Na + flow results in depolarizing changes in resting membrane potential [36–38]. This means that axonal excitability has increased after hdPB treatment and the current influx required to elicit action potentials and ensure nerve conduction was lower. The expression of Na_V_ channels (Na_V_ 1.2 and Na_V_ 1.6) at the origin of persistent Na + currents was shown to be modified in demyelinated axons in the context of MS [38]. Therefore, our results suggest that a treatment with hdPB could generate a phenomenon of nerve plasticity at the axonal level, a potential therapeutic target of nerve excitability in demyelinating neuropathies.

Motor and sensory deficits, as assessed by the MRC total score and the INCAT sensory sum score, improved after treatment. The median distance in the 6-min walking test increased by 69 m, and other clinical and posturography parameters improved. The walking capacity increased by more than 0.5 standard deviations, which is considered a clinically meaningful difference [39]. Although improvement in walking capacities in an open-label, uncontrolled study may be related to important bias, the positive correlation with changes in a nerve excitability parameter (SDTC) that is related to the intrinsic properties of the nerve fibers reinforces the hypothesis of a real effect of the drug. In the context of MS treatment with hdPB, whose initially reported beneficial effects have not been confirmed [9], we did not observe changes in synaptic excitability in the evaluation of the central neural networks [41]. This obviously does not exclude the possibility of excitability changes following hdPB intake at the level of peripheral axons.

Overall, the treatment with hdPB was well tolerated in the 15 patients included in this study. An 80-year-old patient developed an inflammatory encephalopathy with a relapse 428 days after treatment interruption. Although the occurrence of an autoimmune process is surprising at this age [40–42], the relapse that occurred more than one year after drug discontinuation precludes any direct relationship with the treatment. Moreover, in a previous study of hdPB in MS, no central nervous system inflammatory event was observed [9, 12, 43]. Thus, in this study, no new adverse events of hdPB treatment were observed, apart from those already known and reported [7].

The present study has several limitations. First, this is not a placebo-controlled trial, and the positive clinical results may be in part related to a placebo effect, even if they are reinforced by the neurophysiological changes. The number of patients included in the study was small, and a larger study will be required to validate the present observations. Moreover, the population was not homogeneous, with different mechanisms of myelin alterations between the patient groups.

## Conclusions

The present results, especially nerve excitability changes and clinically meaningful improvement in currently less treatable or untreatable conditions represented by anti-MAG neuropathy and CMT1a support the construction of a large randomized controlled trial to assess the potential benefit of treating demyelinating neuropathies with hdPB.

### Supplementary Information

**Additional file 1:** **eTable 1.** Compliance to IP During the Whole Study - SS population. **eTable 2.** Treatment-emergent Adverse Events (TEAEs). **eTable 2a.** Overview of the incidence of TEAEs - SS Population. **eTable 2b.** Display of Adverse Events. **eTable 2c.** IP-related TEAEs by SOC and PT – SS Population. **eTable 2d.** TEAEs by IP Discontinuation by SOC and PT  – SS Population. **eTable 2e.** TEAEs Leading to Death by SOC and PT – SS Population. **eTable 2f.** Other Serious Adverse Events by SOC and PT – SS Population.

## Data Availability

The datasets used and/or analysed during the current study are available from the corresponding author on reasonable request.

## Abbreviations

ATPase: Adenosine 5′-triphosphatase
CIDP: Chronic inflammatory demyelinating polyradiculoneuropathy
CMT: Charcot-Marie-Tooth
COP: Center of pressure
FAS: Full analysis set
HdPB: High-dose pharmaceutical-grade biotin
IEC/IRB: Independent Ethics Committee
INCAT: Inflammatory Neuropathy Cause and Treatment
LOCF: Last observation carried forward
MAG: Myelin-associated glycoprotein
MPZ: Myelin protein 0
MRC: Medical Research Council
MS: Multiple sclerosis
ONLS: Overall Neuropathy Limitations Scale (ONLS)
SDTC: Strength-duration time constant
TEAEs: Treatment-emergent adverse events
