# Supplementary material for: High-dose pharmaceutical-grade biotin in patients with demyelinating neuropathies: a phase 2b open label, uncontrolled, pilot study
Source: BMC Neurol. 2023 Oct 30;23:389. doi: 10.1186/s12883-023-03440-y (PMC10614347; doi:10.1186/s12883-023-03440-y)
Supplement: Supplementary file 1 — Additional file 1: eTable 1. Compliance to IP During the Whole Study - SS population. eTable 2. Treatment-emergent Adverse Events (TEAEs). eTable 2a. Overview of the incidence of TEAEs - SS Population. eTable 2b. Display of Adverse Events. eTable 2c. IP-related TEAEs by SOC and PT – SS Population. eTable 2d. TEAEs by IP Discontinuation by SOC and PT – SS Population. eTable 2e. TEAEs Leading to Death by SOC and PT – SS Population. eTable 2f. Other Serious Adverse Events by SOC and PT – SS Population. [file 12883_2023_3440_MOESM1_ESM.docx]

High-dose pharmaceutical-grade biotin in patients with demyelinating neuropathies

A phase 2b open label, uncontrolled, pilot study

**Online Only Supplements**

**Online Only Text**

Etiological investigation included of the neuropathies included : RBC, haemoglobin, mean corpuscular volume, WBC, platelets, electrolytes (Na, K, HCO3, Ca), protein, creatinine, glomerular filtration rate, fasting blood glucose, AST, ALT, total and free bilirubin, gamma glutamyl transferase, alkaline phosphatase, triglycerides, cholesterol, PT, aPTT, INR2 Biological aetiology additional panels: Serum protein electrophoresis, IgA measurement, HbA1c, HBsAg, HIV, HCV tests, fibrinogen

**Online Only Tables**

eTable 1: Compliance to IP During the Whole Study - SS population

|  | **Statistics** | **CIDP (N=5)** | **anti-MAG (N=5)** | **CMT1 (N=5)** | **All (N=15)** |
| --- | --- | --- | --- | --- | --- |
| Actual treatment duration (week) | N | 5 | 5 | 5 | 15 |
|  | Mean (SD) | 52.26 (2.67) | 47.66 (10.13) | 52.71 (1.36) | 50.88 (6.12) |
|  | Median (IQR) | 53.14 (53.14; 53.14) | 52.14 (50.14; 52.14) | 52.14 (52.14; 52.14) | 52.14 (52.00; 53.14) |
| If treatment interruption, cumulative of interrupted days (day) | N | 0 | 1 | 1 | 2 |
|  | Mean (SD) | NA (NA) | 150.0 (NA) | 1.0 (NA) | 75.5 (105.4) |
|  | Median (IQR) | NA | 150.0 (150.0; 150.0) | 1.0 (1.0; 1.0) | 75.5 (1.0; 150.0) |
| Compliance (%) | N | 5 | 5 | 5 | 15 |
|  | Mean (SD) | 92.06 (5.92) | 90.03 (12.69) | 92.59 (6.33) | 91.56 (8.29) |
|  | Median (IQR) | 93.40 (90.57; 94.52) | 93.30 (90.75; 98.90) | 93.41 (91.52; 97.25) | 93.40 (90.57; 98.35) |

Actual treatment duration (week) = [date of the last IP intake – date of first IP intake + 1)/7] – (cumulative number of interrupted days/7).

Compliance (%) = Actual number of capsules taken/Theoretical number of capsules taken during the actual extent of exposure) x 100

The median (Q1; Q3) of the actual treatment duration was 52.14 (52.00; 53.14) weeks overall and 52.14 (52.14; 52.14) weeks in the CMT1 group, in conformity with the expected treatment duration of 52 weeks, as 3 months were actually scheduled between 2 visits (instead of 12 weeks planned in the protocol).

Median (Q1; Q3) compliance of 93.40 (90.57; 98.35) % overall and of 93.41 (91.52; 97.25) % in the CMT1 group was adequate.

Patient # AH-06 in the anti-MAG group, with a short IP administration duration of 29.7 weeks reported as a major deviation, presented a 150 days IP administration interruption, and a resulting low compliance of 68.3%.

The compliance was never at 100% because the actual date of the last IP intake was not registered, so that, by convention, the day before the last visit was reported in the CRF as the date of the last IP intake. Moreover, 12 weeks were planned by the protocol between 2 visits (with a total IP intake duration of 48 weeks), whereas 3 months were actually scheduled between 2 visits (leading to 52 weeks between first IP intake and last visit for most patients). Patients who completed the study were dispensed a total of 1080 capsules, which covered at maximum 51 weeks and 3 days of treatment. Hence the patients did not have enough IP until the last visit.

eTable 2. Treatment-emergent Adverse Events (TEAEs)

eTable 2a: Overview of the incidence of TEAEs - SS Population

|  | **CIDP (N=5)** | | **anti-MAG (N=5)** | | **CMT1 (N=5)** | | **All (N=15)** | |
| --- | --- | --- | --- | --- | --- | --- | --- | --- |
|  |  | |  | |  | |  | |
|  | **E** | **N (%)** | **E** | **N (%)** | **E** | **N (%)** | **E** | **N (%)** |
| **All AEs** | 15 | 5 (100%) | 10 | 4 (80.0%) | 17 | 5 (100%) | 42 | 14 (93.3%) |
| TEAEs | 15 | 5 (100%) | 10 | 4 (80.0%) | 17 | 5 (100%) | 42 | 14 (93.3%) |
|  | | | | | | | | |
| **Intensity of TEAEs** |  |  |  |  |  |  |  |  |
| Mild | 10 | 5 (100%) | 8 | 3 (60.0%) | 8 | 4 (80.0%) | 26 | 12 (80.0%) |
| Moderate | 4 | 2 (40.0%) | 1 | 1 (20.0%) | 8 | 3 (60.0%) | 13 | 6 (40.0%) |
| Severe | 1 | 1 (20.0%) | 1 | 1 (20.0%) | 1 | 1 (20.0%) | 3 | 3 (20.0%) |
|  | | | | | | | | |
| **Serious TEAEs** | 1 | 1 (20.0%) | 1 | 1 (20.0%) | 0 | 0 | 2 | 2 (13.3%) |
|  | | | | | | | | |
| **IP-related TEAEs*** | 5 | 3 (60.0%) | 0 | 0 | 5 | 3 (60.0%) | 10 | 6 (40.0%) |
|  | | | | | | | | |
| **IP-related serious TEAEs*** | 0 | 0 | 0 | 0 | 0 | 0 | 0 | 0 |
|  | | | | | | | | |
| **TEAEs leading to IP discontinuation** | 1 | 1 (20.0%) | 0 | 0 | 0 | 0 | 1 | 1 (6.7%) |
|  | | | | | | | | |
| **TEAEs leading to death** | 1 | 1 (20.0%) | 0 | 0 | 0 | 0 | 1 | 1 (6.7%) |
|  | | | | | | | | |
| **Serious TEAEs leading to IP discontinuation** | 1 | 1 (20.0%) | 0 | 0 | 0 | 0 | 1 | 1 (6.7%) |
|  |  |  |  |  |  |  |  |  |
| E = Number of events - N = Number of Patients - % = (number of Patients / number of Patients by group) x 100 | | | | | | | | |

*Related TEAEs if the causality recorded in the CRF is Definite, Probable, Likely, Possible, Conditional or missing

Overall, 14 patients (93.3%) presented 42 AEs. In the CMT1 group, 5 patients (100%)

### eTable 2b Display of Adverse Events

TEAEs by SOC and PT – SS Population. Summary of TEAEs by SOCs and PT. Only SOCs encompassing events reported by more than 1 patient are displayed.

|  | **CIDP (N=5)** | | **anti-MAG (N=5)** | | **CMT1 (N=5)** | | **All (N=15)** | |
| --- | --- | --- | --- | --- | --- | --- | --- | --- |
| **System organ class preferred term** | **E** | **N (%)** | **E** | **N (%)** | **E** | **N (%)** | **E** | **N (%)** |
| At least one TEAE | 15 | 5 (100%) | 10 | 4 (80.0%) | 17 | 5 (100%) | 42 | 14 (93.3%) |
|  | | | | | | | | |
| **Nervous system disorders** | **4** | **3 (60.0%)** | **3** | **2 (40.0%)** | **4** | **4 (80.0%)** | **11** | **9 (60.0%)** |
| Insomnia | 2 | 2 (40.0%) | 0 | 0 | 1 | 1 (20.0%) | 3 | 3 (20.0%) |
| Autoimmune encephalopathy | 1 | 1 (20.0%) | 0 | 0 | 0 | 0 | 1 | 1 (6.7%) |
| Balance disorder | 0 | 0 | 1 | 1 (20.0%) | 0 | 0 | 1 | 1 (6.7%) |
| Memory impairment | 1 | 1 (20.0%) | 0 | 0 | 0 | 0 | 1 | 1 (6.7%) |
| Muscle contractions involuntary | 0 | 0 | 0 | 0 | 1 | 1 (20.0%) | 1 | 1 (6.7%) |
| Neuralgia | 0 | 0 | 2 | 1 (20.0%) | 0 | 0 | 2 | 1 (6.7%) |
| Restless legs syndrome | 0 | 0 | 0 | 0 | 1 | 1 (20.0%) | 1 | 1 (6.7%) |
| Sciatica | 0 | 0 | 0 | 0 | 1 | 1 (20.0%) | 1 | 1 (6.7%) |
|  | | | | | | | | |
| **Musculoskeletal and connective tissue disorders** | **3** | **2 (40.0%)** | **0** | **0** | **4** | **3 (60.0%)** | **7** | **5 (33.3%)** |
| Arthralgia | 0 | 0 | 0 | 0 | 4 | 3 (60.0%) | 4 | 3 (20.0%) |
| Muscle spasms | 1 | 1 (20.0%) | 0 | 0 | 0 | 0 | 1 | 1 (6.7%) |
| Musculoskeletal stiffness | 1 | 1 (20.0%) | 0 | 0 | 0 | 0 | 1 | 1 (6.7%) |
| Pain in extremity | 1 | 1 (20.0%) | 0 | 0 | 0 | 0 | 1 | 1 (6.7%) |
|  | | | | | | | | |
| **General disorders and administration site conditions** | **1** | **1 (20.0%)** | **1** | **1 (20.0%)** | **2** | **2 (40.0%)** | **4** | **4 (26.7%)** |
| Fatigue | 1 | 1 (20.0%) | 1 | 1 (20.0%) | 1 | 1 (20.0%) | 3 | 3 (20.0%) |
| Oedema peripheral | 0 | 0 | 0 | 0 | 1 | 1 (20.0%) | 1 | 1 (6.7%) |
|  | | | | | | | | |
| **Gastrointestinal disorders** | **1** | **1 (20.0%)** | **1** | **1 (20.0%)** | **2** | **1 (20.0%)** | **4** | **3 (20.0%)** |
| Diarrhoea | 0 | 0 | 0 | 0 | 1 | 1 (20.0%) | 1 | 1 (6.7%) |
| Gastric disorder | 0 | 0 | 1 | 1 (20.0%) | 0 | 0 | 1 | 1 (6.7%) |
| Nausea | 1 | 1 (20.0%) | 0 | 0 | 0 | 0 | 1 | 1 (6.7%) |
| Vomiting | 0 | 0 | 0 | 0 | 1 | 1 (20.0%) | 1 | 1 (6.7%) |
|  |  |  |  |  |  |  |  |  |
| **Injury, poisoning and procedural complications** | **0** | **0** | **2** | **2 (40.0%)** | **1** | **1 (20.0%)** | **3** | **3 (20.0%)** |
| Ankle fracture | 0 | 0 | 0 | 0 | 1 | 1 (20.0%) | 1 | 1 (6.7%) |
| Burn oesophageal | 0 | 0 | 1 | 1 (20.0%) | 0 | 0 | 1 | 1 (6.7%) |
| Foot fracture | 0 | 0 | 1 | 1 (20.0%) | 0 | 0 | 1 | 1 (6.7%) |
|  | | | | | | | | |
| **Investigations** | **1** | **1 (20.0%)** | **1** | **1 (20.0%)** | **1** | **1 (20.0%)** | **3** | **3 (20.0%)** |
| Laboratory test interference | 1 | 1 (20.0%) | 0 | 0 | 1 | 1 (20.0%) | 2 | 2 (13.3%) |
| Haemoglobin decreased | 0 | 0 | 1 | 1 (20.0%) | 0 | 0 | 1 | 1 (6.7%) |
|  | | | | | | | | |
| **Skin and subcutaneous tissue disorders** | **2** | **1 (20.0%)** | **1** | **1 (20.0%)** | **1** | **1 (20.0%)** | **4** | **3 (20.0%)** |
| Pruritus | 0 | 0 | 1 | 1 (20.0%) | 1 | 1 (20.0%) | 2 | 2 (13.3%) |
| Alopecia | 1 | 1 (20.0%) | 0 | 0 | 0 | 0 | 1 | 1 (6.7%) |
| Rash popular | 1 | 1 (20.0%) | 0 | 0 | 0 | 0 | 1 | 1 (6.7%) |
| E = number of events; n = number of patients; % = (number of patients / number of patients by group) x 100. | | | | | | | | |

The most frequently represented SOCs were:

- Nervous system disorders in 9 (60.0%) patients overall
- Musculoskeletal and connective tissue disorders in 5 (33.3%) patients overall,
- General disorders and administration site conditions in 4 (26.7%) patients overall,
- Gastro-intestinal disorders in 3 (20.0%) of patients overall,
- Injury, poisoning and procedural complications in 3 (20.0%) patients overall,
- Investigations in 3 (20.0%) of patients overall,
- Skin and subcutaneous tissue disorders in 3 (20.0%) patients overall.

Other SOCs encompassed events reported in only 1 (6.7%) patient overall, and were infections and infestations, neoplasms benign, malignant and unspecified (incl cysts and polyps), psychiatric disorders, respiratory, thoracic and mediastinal disorders, surgical and medical procedures and vascular disorders

#### TEAEs by PT

The most frequently reported TEAEs by PT were:

- Insomnia in 3 (20.0%) patients overall,
- Arthralgia in 3 (20.0%) patients overall,
- Fatigue in 3 (20.0%) patients overall,
- Laboratory test interference in 2 (13.3%) patients overall,
- Pruritus in 2 (13.3%) patients overall.

Laboratory test interferences due to High-dose pharmaceutical-grade biotin were reported in 2 (13.3%) patients overall.

Other TEAEs were reported in 1 (6.7%) patient overall.

### Analysis of Adverse Events

### eTable 2c IP-related TEAEs by SOC and PT – SS Population

|  | **CIDP (N=5)** | | **anti-MAG (N=5)** | | **CMT1 (N=5)** | | **All (N=15)** | |
| --- | --- | --- | --- | --- | --- | --- | --- | --- |
| **System Organ Class Preferred Term** | **E** | **N (%)** | **E** | **N (%)** | **E** | **N (%)** | **E** | **N (%)** |
| At least one IP related TEAEs* | 5 | 3 (60.0%) | 0 | 0 | 5 | 3 (60.0%) | 10 | 6 (40.0%) |
|  | | | | | | | | |
| **Nervous system disorders** | **1** | **1 (20.0%)** | **0** | **0** | **2** | **2 (40.0%)** | **3** | **3 (20.0%)** |
| Insomnia | 1 | 1 (20.0%) | 0 | 0 | 1 | 1 (20.0%) | 2 | 2 (13.3%) |
| Muscle contractions involuntary | 0 | 0 | 0 | 0 | 1 | 1 (20.0%) | 1 | 1 (6.7%) |
|  | | | | | | | | |
| **Investigations** | **1** | **1 (20.0%)** | **0** | **0** | **1** | **1 (20.0%)** | **2** | **2 (13.3%)** |
| Laboratory test interference | 1 | 1 (20.0%) | 0 | 0 | 1 | 1 (20.0%) | 2 | 2 (13.3%) |
|  | | | | | | | | |
| **Skin and subcutaneous tissue disorders** | **2** | **1 (20.0%)** | **0** | **0** | **1** | **1 (20.0%)** | **3** | **2 (13.3%)** |
| Alopecia | 1 | 1 (20.0%) | 0 | 0 | 0 | 0 | 1 | 1 (6.7%) |
| Pruritus | 0 | 0 | 0 | 0 | 1 | 1 (20.0%) | 1 | 1 (6.7%) |
| Rash popular | 1 | 1 (20.0%) | 0 | 0 | 0 | 0 | 1 | 1 (6.7%) |
|  | | | | | | | | |
| **General disorders and administration site conditions** | **0** | **0** | **0** | **0** | **1** | **1 (20.0%)** | **1** | **1 (6.7%)** |
| Fatigue | 0 | 0 | 0 | 0 | 1 | 1 (20.0%) | 1 | 1 (6.7%) |
|  | | | | | | | | |
| **Musculoskeletal and connective tissue disorders** | **1** | **1 (20.0%)** | **0** | **0** | **0** | **0** | **1** | **1 (6.7%)** |
| Muscle spasms | 1 | 1 (20.0%) | 0 | 0 | 0 | 0 | 1 | 1 (6.7%) |
|  | | | | | | | | |
| E = Number of events; N = Number of Patients; % = (number of Patients / number of Patients by group) x 100. | | | | | | | | |

*Related TEAEs if the causality recorded in the CRF is Definite, Probable, Likely, Possible, Conditional or missing

Six (40%) patients overall presented a total of 10 IP-related TEAEs.

The SOCs with the most frequently reported IP-related TEAEs were:

- Nervous system disorders in 20.0% of patients,
- Investigations in 13.3% of patients,
- Skin and subcutaneous tissue disorders in 13.3% of patients,
- General disorders and administration site conditions in 6.7% of patients,
- Musculoskeletal and connective tissue disorders in 6.7% of patients.

The IP-related TEAEs were:

- Insomnia in 2 (13.3%) patients;
- Muscle contractions involuntary 1 (6.7%) patient,
- Laboratory test interference in 2 (13.3%) patients,
- Alopecia in 1 (6.7%) patient,
- Pruritus in 1 (6.7%) patient,
- Rash papular in 1 (6.7%) patient,
- Fatigue in 1 (6.7%) patient,
- Muscle spams in 1 (6.7%) patient.

No IP-related TEAEs was of severe intensity and none was considered as serious. All were resolved by the end of the study.

#### eTable 2d TEAEs by IP Discontinuation by SOC and PT – SS Population

|  | **CIDP (N=5)** | | **anti-MAG (N=5)** | | **CMT1 (N=5)** | | **All (N=15)** | |
| --- | --- | --- | --- | --- | --- | --- | --- | --- |
| **System Organ Class Preferred Term** | **E** | **N (%)** | **E** | **N (%)** | **E** | **N (%)** | **E** | **N (%)** |
| At least one TEAEs leading to IP discontinuation | 1 | 1 (20.0%) | 0 | 0 | 0 | 0 | 1 | 1 (6.7%) |
|  | | | | | | | | |
| Nervous system disorders | 1 | 1 (20.0%) | 0 | 0 | 0 | 0 | 1 | 1 (6.7%) |
| Autoimmune encephalopathy | 1 | 1 (20.0%) | 0 | 0 | 0 | 0 | 1 | 1 (6.7%) |
|  | | | | | | | | |
| E = Number of events; N = Number of Patients;  % = (number of Patients / number of Patients by group) x 100. | | | | | | | | |

There was 1 TEAE leading to IP discontinuation: serious and severe autoimmune encephalopathy in 1 CIDP patient. This TEAE was considered as not IP-related by the investigator.

eTable 2e: TEAEs Leading to Death by SOC and PT – SS Population

|  | **CIDP (N=5)** | | **anti-MAG (N=5)** | | **CMT1 (N=5)** | | **All (N=15)** | |
| --- | --- | --- | --- | --- | --- | --- | --- | --- |
| **System Organ Class Preferred Term** | **E** | **N (%)** | **E** | **N (%)** | **E** | **N (%)** | **E** | **N (%)** |
| At least one TEAEs leading to death | 1 | 1 (20.0%) | 0 | 0 | 0 | 0 | 1 | 1 (6.7%) |
|  | | | | | | | | |
| **Nervous system disorders** | **1** | **1 (20.0%)** | **0** | **0** | **0** | **0** | **1** | **1 (6.7%)** |
| Autoimmune encephalopathy | 1 | 1 (20.0%) | 0 | 0 | 0 | 0 | 1 | 1 (6.7%) |
|  | | | | | | | | |
| E = Number of events; N = Number of Patients;  % = (number of Patients / number of Patients by group) x 100.  S | | | | | | | | |

There was one TEAE leading to death: serious and severe autoimmune encephalopathy in CIDP patient (# OB-04), which lead to death 15 months after IP withdrawal. This TEAE was considered as not IP-related by the investigator.

####

#### eTable 2f Other Serious Adverse Events by SOC and PT – SS Population

|  | **CIDP (N=5)** | | **anti-MAG (N=5)** | | **CMT1 (N=5)** | | **All (N=15)** | |
| --- | --- | --- | --- | --- | --- | --- | --- | --- |
| **System Organ Class Preferred Term** | **E** | **N (%)** | **E** | **N (%)** | **E** | **N (%)** | **E** | **N (%)** |
| At least one Serious TEAEs | 1 | 1 (20.0%) | 1 | 1 (20.0%) | 0 | 0 | 2 | 2 (13.3%) |
|  | | | | | | | | |
| **Neoplasms benign, malignant and unspecified (incl cysts and polyps)** | **0** | **0** | **1** | **1 (20.0%)** | **0** | **0** | **1** | **1 (6.7%)** |
| Clear cell renal cell carcinoma | 0 | 0 | 1 | 1 (20.0%) | 0 | 0 | 1 | 1 (6.7%) |
|  | | | | | | | | |
| **Nervous system disorders** | **1** | **1 (20.0%)** | **0** | **0** | **0** | **0** | **1** | **1 (6.7%)** |
| Autoimmune encephalopathy | 1 | 1 (20.0%) | 0 | 0 | 0 | 0 | 1 | 1 (6.7%) |
|  | | | | | | | | |
| E = Number of events; N = Number of Patients;  % = (number of Patients / number of Patients by group) x 100. | | | | | | | | |

Besides the autoimmune encephalopathy event leading to death 15 months after IP withdrawal, there was another serious TEAE: a clear cell renal cell carcinoma in 1 anti-MAG patient (# PG-08) from which the patient recovered. This TEAE was considered as not IP-related by the investigator and did not lead to IP change.

One serious TEAE consisted of an autoimmune encephalopathy with tumefactive lesions in 1 CIDP patient, which led to IP withdrawal. A relapse occurred 428 days after IP discontinuation leading to death shortly after. The relapse 15 months after IP discontinuation precluded any relation with it ;

One renal clear cells carcinoma diagnosed on the pathological examination of a planned robot-assisted right partial nephrectomy in 1 anti-MAG patient which did not lead to dose changes and from which the patient recovered through surgery. The presence of a kidney abnormality on a imaging investigation prior inclusion, precludes any link with the IP.

A third severe AE was a left external malleolar fracture in 1 CMT1 patient which did not lead to dose changes and from which the patient recovered.
